# Neutrophil-to-Lymphocyte Ratio Is Associated With Circumferential Wall Enhancement of Unruptured Intracranial Aneurysm

**DOI:** 10.3389/fneur.2022.879882

**Published:** 2022-05-20

**Authors:** Xiao-Bing Wu, Jing-Lian Zhong, Sheng-Wen Wang, Yun Su, Pei-Sheng Chen, Zhong-Jun Li, Chun Xiang, Wang-Qing Cai, Zhong-Song Shi

**Affiliations:** ^1^Department of Neurosurgery, Sun Yat-sen Memorial Hospital, Sun Yat-sen University, Guangzhou, China; ^2^Department of Radiology, Sun Yat-sen Memorial Hospital, Sun Yat-sen University, Guangzhou, China

**Keywords:** intracranial aneurysm, neutrophil-lymphocyte ratio, vessel wall imaging, aneurysm wall enhancement, blood marker

## Abstract

**Background and Purpose:**

Neutrophil-lymphocyte ratio (NLR) predicts clinical outcomes in patients with stroke. Aneurysm wall enhancement (AWE) on high-resolution vessel wall magnetic resonance imaging (HR-VWI) is an inflammation marker for intracranial aneurysm (IA). This study aims to evaluate the association of NLR as a peripheral blood inflammatory marker with circumferential AWE in patients with IA.

**Methods:**

We analyzed data of consecutive patients harboring IAs between September 2017 and December 2021 at our institution. The peripheral blood inflammatory indicators were compared between patients with ruptured and unruptured IAs. The presence of circumferential AWE in unruptured IA was identified and quantitatively measured using the aneurysm-to-pituitary stalk contrast ratio (CRstalk) on HR-VWI. We used the optimal cutoff value of 0.5 for CRstalk to differentiate circumferential AWE in unruptured IAs. We assessed the relationship of clinical, laboratory, and radiological characteristics with circumferential AWE and CRstalk ≥0.5 in unruptured IAs.

**Results:**

The study group was composed of one hundred and twenty-five patients with 142 IAs. NLR level at admission was significantly higher in patients with ruptured IAs than those with unruptured IAs (7.55 vs. 1.81; *P* < 0.001). AWE on HR-VWI was present in 30 patients with unruptured IAs (38.5%), including 12 with focal AWE and 18 with circumferential AWE. NLR (odds ratio (OR), 2.168; 95% CI, 1.149–4.088) and size (odds ratio, 1.370; 95% CI, 1.126–1.667) were independently associated with circumferential AWE in unruptured IA. NLR was also independently associated with circumferential AWE in small unruptured IA (<7 mm). Furthermore, NLR level at admission was associated with CRstalk ≥.5 in patients with unruptured IA. The optimal cutoff value of NLR for circumferential AWE was 1.86.

**Conclusion:**

NLR is a valuable peripheral blood inflammatory marker is more often in the rupture status of IA and was associated with circumferential AWE on HR-VWI in unruptured IA.

## Introduction

Intracranial aneurysms (IA) are the leading cause of subarachnoid hemorrhage (SAH) in the general population, resulting in a high mortality and disability rate once ruptured. An increased risk of rupture occurs in unruptured IA after detecting aneurysm growth without preventive neurosurgical or endovascular treatment (1, 2). Inflammation cascade and biomechanical remodeling in the aneurysm wall are critical to the formation and progression of IA and associated with aneurysm rupture from unruptured IA (3–5). High-resolution vessel wall imaging (HR-VWI) is increasingly used as a valuable non-invasive imaging tool to understand the symptoms, rupture, growth risk of IA and guide the effective management strategy for patients with unruptured IA in the clinical setting (6–12). More evidence recently suggests that aneurysm wall enhancement (AWE) on HR-VWI in unruptured IA may correlate with inflammatory cell invasion and atherosclerosis, neovascularization, and vasa vasorum in the aneurysm wall revealed from histological and surgical findings (13–17). AWE on HR-VWI is associated with a localized decrease of plasma concentration of the anti-inflammatory cytokine IL-10 in the aneurysm sac (18, 19). However, the association of peripheral blood inflammatory marker with AWE in patients with unruptured IA remains unclear.

Neutrophil-lymphocyte ratio (NLR) indicates the inflammation status in clinical practice, calculated as the absolute neutrophil count divided by the absolute lymphocyte count in the routine blood test. Elevated NLR predicts poor outcomes in patients with acute ischemic stroke, spontaneous intracerebral hemorrhage, and SAH resulting from ruptured IA (20–23). In this study, we aimed to investigate the association of NLR as a peripheral blood inflammatory marker with AWE on HR-VWI in patients with unruptured IA.

## Materials and Methods

### Patient Selection

All consecutive patients harboring intracranial aneurysms (ruptured and unruptured) who were candidates for initial endovascular and surgical treatment were prospectively maintained in a database between September 2017 and December 2021 at our institution. Patients were older than 18 years old. The presence of an intracranial aneurysm was confirmed by digital subtraction angiography. Patients with ruptured aneurysms underwent brain CT and lumbar puncture to confirm the presence of SAH. Patients with unruptured IA were enrolled in this study that aneurysms can be identified on MR angiography and had images of HR-VWI without artifacts. We exclude patients with a history of pneumonia, heart diseases, autoimmune disease, hematological diseases, cancer, chronic liver, kidney insufficiency, and patients with aneurysms in the extracranial or cavernous sinus of the internal carotid artery. This study was approved by the local institutional review board at our institution.

### Peripheral Blood Test

The peripheral blood test within 24 h after admission was recorded, including white blood cell count, neutrophils, lymphocytes, monocyte counts, high-sensitivity C-reactive protein, mean platelet volume, total cholesterol, triglycerides, low-density lipoprotein cholesterol, high-density lipoprotein cholesterol, apolipoprotein E, and blood calcium. The NLR was calculated as follows: NLR = neutral counts/lymphocyte counts. The peripheral blood was collected from patients with ruptured IA before lumbar puncture.

### HR-VWI Examination

Patients with unruptured IAs underwent brain MR for HR-VWI on a 3.0T MRI scanner with a 32-channel head coil (Achieva TX, Philips Healthcare, Best, the Netherlands). We used our previously published HR-VWI protocol in this study (24).The 2D T1-weighted black-blood vessel wall sequence was performed between September 2017 and September 2018. Since October 2018, the 3D T1-weighted VISTA sequence was performed. Post-contrast 2D/3D T1-weighted images were scanned for 5 min after the injection of 0.1 mmol/kg gadopentetate glucosamine (Gd-DTPA, Hokuriku Pharmaceutical, China). The black-blood pulse with motion-sensitized driven-equilibrium was used in this study which applies a magnetization preparation sequence that causes moving spins to diphase and thereby suppresses signals from blood vessels with sufficient flow.

We divided the patten of AWE of saccular unruptured IAs as none AWE, focal AWE, and circumferential AWE according to the precontrast and postcontrast HR-VWI T1-weighted sequences (6–8).We measured signal intensity (SI) of the aneurysm wall (SIwall) and the pituitary stalk (SIstalk) on the 3D postcontrast HR-VWI T1-weighted sequence. The aneurysm-to-pituitary stalk contrast ratio (CRstalk) was calculated using the maximal SI value of SIwall and SIstalk as CRstalk = SIwall/SIstalk, which was an optimal quantitative analysis of AWE in unruptured IA (24–26).

### Statistical Analysis

The data, including medical history, laboratory examination, and clinical and radiological characteristics, were recorded. Two readers who were blinded to the clinical data independently reviewed the HR-VWI T1-weighted images to identify the presence and pattern of AWE in saccular unruptured IAs. A third reader resolved disagreements. Cohen κ statistics were used to assess the interreader agreement. κ values >0.80 were regarded as excellent for the identification of AWE.

We compared data variables between patients with ruptured aneurysms and those with unruptured IAs. Then, we analyzed the association of these variables with all patterns of AWE and circumferential AWE in all unruptured IAs and the subgroup of unruptured IAs small than 7 mm. We used the optimal cutoff value of 0.5 for CRstalk to differentiate circumferential AWE from non-circumferential AWE in unruptured IA with a 3D HR-VWI T1-weighted sequence (19, 24).Then, we analyzed the association of these variables with CRstalk ≥0.5 in unruptured IAs.

We performed statistical analysis using the SPSS 22 software. Continuous and categorical variables were analyzed by the Student's *t*-test, Mann-Whitney *U*-test, Fisher's exact, or chi-square test. *P*-value < 0.05 was considered statistically significant for the results. Multivariate logistic regression analyses were conducted to determine which factors were independent risk factors for circumferential AWE after adjusting for variables with *P* < 0.05 in the univariate comparisons. The cutoff values for NLR with the best sensitivity and specificity to differentiate circumferential AWE from non-circumferential AWE were identified using the receiver operating characteristic (ROC) curve.

## Results

### Clinical Characteristics

During a four-year study, 179 consecutive patients with 198 IAs were identified, but only 125 patients with 142 IAs were included in this study after excluding those for defined criteria. They were 35 patients with aneurysmal SAH and 90 patients with unruptured IAs. The mean age was 56 ± 11.9 years, and 75 (60%) were women. The mean size of aneurysms was 7.09 ± 6.11 mm (1.3–38 mm). There were thirty-four (23.9%) cases of aneurysms which are located in the posterior circulation. Among 97 patients with unruptured IAs, 78 saccular IAs were identified in 73 patients, and 19 fusiform IAs were from 17 patients. The characteristics of the patients with ruptured and unruptured IAs are shown in Table 1.

**Table 1:** Characteristics of ruptured and unruptured intracranial aneurysms.

	**Total** **(*n* = 125)**	**Unruptured aneurysm** **(*n* = 90)**	**Ruptured aneurysm** **(*n* = 35)**	***P*-value**
Age (yr)	55.98 ± 11.89	55.13 ± 11.81	58.14 ± 11.99	0.205
Female	75 (60%)	48 (53.3%)	27 (77.1%)	0.015
Hypertension	51 (40.8%)	38 (42.2%)	13 (37.1%)	0.604
Diabetes	11 (8.8%)	9 (10.0%)	2 (5.7%)	0.726
Smoking	29 (23.2%)	23 (25.6%)	6 (17.1%)	0.356
Drinking	8 (6.4%)	7 (7.8%)	1 (2.9%)	0.440
Size (mm)	5.1 (3.6–8.2)	5.2 (3.7–8.6)	5.0 (3.3–7.6)	0.110
**Location**				0.347
Anterior circulation	108 (76.1%)	76 (78.4%)	32 (71.1%)	
Posterior circulation	34 (23.9%)	21 (21.6%)	13 (28.9%)	
Irregular shape	62 (43.7%)	34 (35.1%)	28 (62.2%)	0.002
Saccular aneurysm	121 (85.2%)	80 (82.5%)	41 (91.1%)	0.212
Fusiform aneurysm	21 (14.8%)	17 (17.5%)	4 (8.9%)	0.212
White blood cells, ×10^9^/L	7.08 (5.69–9.36)	6.44 (5.04–7.32)	11.21 (9.28–12.58)	<0.001
Lymphocyte counts, ×10^9^/L	1.75 (1.32–2.25)	1.90 (1.90–2.31)	1.21 (0.86–1.80)	<0.001
Neutral counts, ×10^9^/L	5.34 (3.27–6.73)	3.58 (2.87–4.35)	9.08 (6.94–11.28)	<0.001
Monocyte counts, ×10^9^/L	0.45 (0.34–0.62)	0.42 (0.33–0.55)	0.58 (0.35–0.83)	0.008
Neutrophil-to-lymphocyte ratio	2.15 (1.57–4.16)	1.81 (1.47–2.31)	7.55 (3.91–13.30)	<0.001

### NLR and Rupture Status of Aneurysm

The median NLR of the total patients was 2.15 ranging from 0.71 to 18.84. The level of NLR at admission was significantly higher in patients with aneurysmal SAH than in those with unruptured IAs (7.55 vs. 1.81; *P* < 0.001). Aneurysmal SAH patients were more likely to have higher white blood cell counts, neutrophils, monocyte counts, and lower lymphocytes than patients with unruptured IA (*P* < 0.01). Comparisons of the NLR and other laboratory parameters between both groups are shown in Table 1.

### NLR and AWE in Unruptured IA

In 73 patients with 78 saccular IAs, AWE on HR-VWI was present in 30 (38.5%), including 12 unruptured IAs with focal AWE and 18 with circumferential AWE. The interreader agreement for the identification of AWE was excellent, with κ = 0.92.The aneurysm size was significantly larger in patients with AWE than in those without AWE (10.01 mm vs. 4.81 mm, *P* = 0.002). The level of NLR at admission was significantly higher in the group with AWE than in those without AWE (1.98 vs. 1.65, *P* = 0.039). Comparisons of the characteristics of the patients with unruptured IA with and without circumferential AWE are shown in Table 2. The aneurysm size was significantly larger in patients with circumferential AWE than in those without circumferential AWE (8.7vs. 4.6 mm, *P* < 0.001). The level of NLR at admission was significantly higher in the group with circumferential AWE than in those without circumferential AWE (2.16 vs. 1.61, *P* = 0.003). In the multiple logistic regression model, NLR (odds ratio (OR), 2.168; 95% CI, 1.149–4.088, *P* = 0.017) and size (OR, 1.370; 95% CI, 1.126–1.667, *P* = 0.002) were independently associated with circumferential AWE in saccular unruptured IAs (Table 3).

**Table 2:** Characteristics of unruptured intracranial aneurysms with and without circumferential aneurysm wall enhancement.

	**All UIA patients** **(*n* = 73)**	**UIA patients with CAWE** **(*n* = 18)**	**UIA patients with non-CAWE** **(*n* = 55)**	***P*-value**
Age (yr)	56.15 ± 11.1	56.11 ± 14.47	56.16 ± 9.93	0.986
Female	44 (60.3%)	11 (61.1%)	33 (60%)	0.933
Hypertension	32 (43.8%)	11 (61.1%)	21 (38.2%)	0.089
Diabetes	8 (11.0%)	2 (11.1%)	6 (10.9%)	1.000
Smoking	17 (23.3%)	6 (33.3%)	11 (20%)	0.201
Drinking	4 (5.5%)	2 (11.1%)	2 (3.6%)	0.206
Size	5.1 (3.8–7.9)	8.7 (5.4–14.4)	4.6 (3.7–6.5)	<0.001
**Location**				0.211
Anterior circulation	64 (87.7%)	14 (77.8%)	50 (90.9%)	
Posterior circulation	9 (12.3%)	4 (22.2%)	5 (9.1%)	
Irregular shape	24 (32.9%)	9 (50%)	15 (27.3%)	0.075
White blood cells, ×10^9^/L	6.26 (4.82–7.19)	6.80 (6.06–7.23)	6.00 (4.60–7.18)	0.077
Lymphocyte counts, ×10^9^/L	1.83 (1.59–2.36)	1.72 (1.54–2.19)	1.95 (1.59–2.38)	0.255
Neutral counts, ×10^9^/L	3.51 (2.82–4.22)	4.07 (3.42–4.41)	3.40 (2.42–3.93)	0.009
Monocyte counts, ×10^9^/L	0.41 (0.33–0.55)	0.46 (0.41–0.58)	0.39 (0.32–0.54)	0.187
NLR	1.75 (1.45–2.30)	2.16 (1.88–2.75)	1.61 (1.31–2.13)	0.003
high-sensitivity C-reactive protein, mg/L	0.90 (0.56–1.88)	1.01 (0.78–4.35)	0.78 (0.39–1.82)	0.079
Total cholesterol, mmol/L	4.73 (4.04–5.39)	4.69 (3.80–5.29)	4.85 (4.10–5.39)	0.282
Triglycerides, mmol/L	1.24 (0.95–1.56)	1.24 (0.98–1.66)	1.24 (0.93–1.56)	0.798
High-density lipoprotein cholesterol, mmol/L	1.13 (0.98–1.38)	1.05 (0.88–1.30)	1.15 (1.02–1.40)	0.058
Low-density lipoprotein cholesterol, mmol/L	2.91 (2.50–3.45)	2.73 (2.32–3.36)	2.99 (2.54–3.45)	0.288
Apolipoprotein E, mg/L	35.5 (28.05–42.4)	37.05 (29.78–41.88)	34.4 (27.60–42.8)	0.883

*CAWE, circumferential aneurysm wall enhancement; NLR, neutrophil-to-lymphocyte ratio; UIA, unruptured intracranial aneurysm*.

**Table 3:** Multiple logistic regression analysis for circumferential aneurysm wall enhancement in the unruptured intracranial aneurysm.

	**All patients with UIA (*****n*** **=** **73)**		**Patients with UIA smaller than 7 mm (*****n*** **=** **52)**	
	**Odds ratio (95% CI)**	***P*-value**	**Odds ratio (95% CI)**	***P-*value**
NLR	2.168 (1.149–4.088)	0.017	2.712 (1.174–6.264)	0.019
Size	1.370 (1.126–1.667)	0.002	—	NS
Age	—	NS	1.140 (1.018–1.277)	0.024

*CI, indicates confidence interval; NLR, neutrophil-to-lymphocyte ratio; NS, not significant; UIA, unruptured intracranial aneurysm*.

In the subgroup with unruptured IAs smaller than 7 mm, the level of NLR at admission was also significantly higher in the group with circumferential AWE than those without circumferential AWE (2.70 vs. 1.66, *P* = 0.005). Furthermore, NLR (OR, 2.712; 95% CI, 1.174–6.264, *P* = 0.019) and age (OR, 1.140; 95% CI, 1.018–1.277, *P* = 0.024) were independently associated with circumferential AWE in saccular unruptured IAs smaller than 7 mm (Table 3).

**Figure 1:**
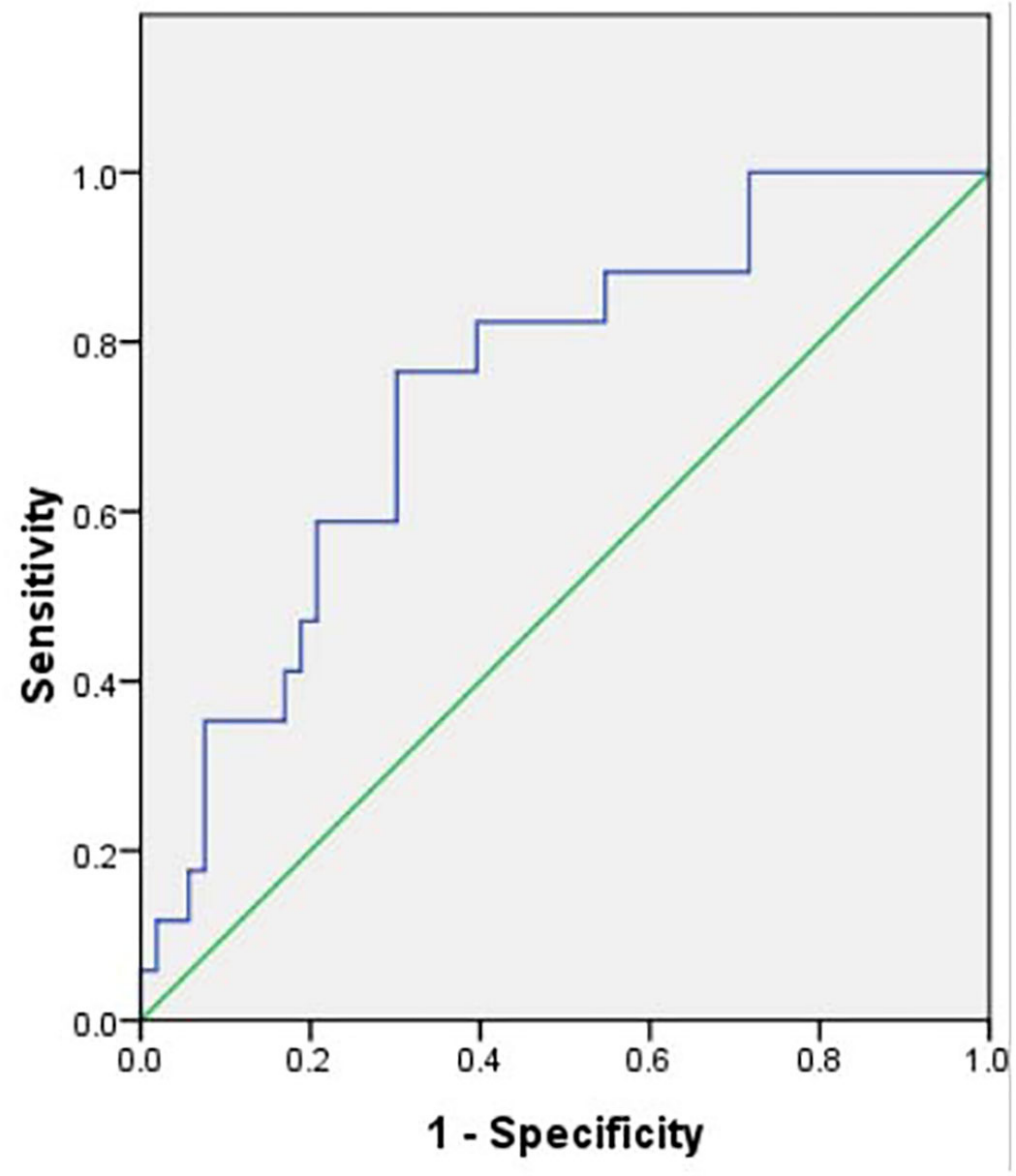
Receiver operating characteristic curve of neutrophil-to-lymphocyte ratio to differentiate circumferential aneurysm wall enhancement, the area under the curve was 0.734. The cutoff value of the neutrophil-to-lymphocyte ratio was 1.86.

The ROC curve indicated that the most reliable cutoff value of NLR to differentiate circumferential AWE from non-circumferential AWE was 1.86, the AUC was 0.734 (Figure 1). When the cutoff value of NLR was 1.86, the sensitivity and specificity were 0.778 and 0.673, respectively.

### NLR and CRstalk in Unruptured IA

**Figure 2:**
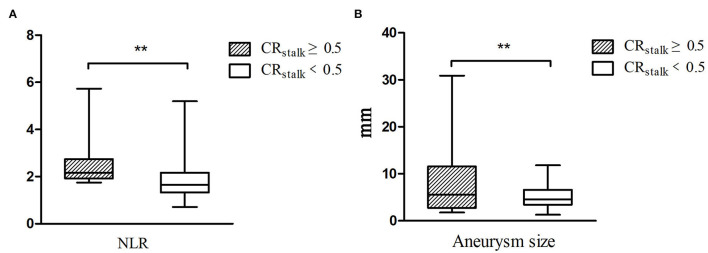
The neutrophil-to-lymphocyte ratio (NLR) and aneurysm size were associated with CRstalk in patients with unruptured intracranial aneurysms. The level of NLR at admission was higher in the group with CRstalk ≥0.5 **(A)**, and the aneurysm size was larger in the group with CRstalk ≥0.5 **(B)**. **, *p* < 0.01.

A total of 57 patients with 60 UIAs were scanned by a 3D HR-VWI sequence to quantify the CRstalk values in this study. Amon the said patients, 11 UIAs in 11 patients had the value of CRstalk ≥0.5, and 49 UIAs in 46 patients had the value of CRstalk <0.5. The aneurysm size was significantly larger in the group with CRstalk ≥0.5 than those with CRstalk <0.5 (6.9 mm vs. 4.6 mm, *P* = 0.001). The level of NLR at admission was significantly higher in the group with CRstalk ≥0.5 than those with CRstalk <0.5 (2.17 vs. 1.66, *P* = 0.007) (Figure 2).

## Discussion

Our study showed that NLR level at admission was significantly higher in patients with ruptured IAs than those with unruptured IAs. In patients with unruptured IA, elevated NLR was independently associated with circumferential AWE on HR-VWI, and this was still true for analyses in unruptured IA smaller than 7 mm. NLR level at admission was associated with CRstalk ≥0.5 in patients with unruptured IA. The optimal cutoff value of NLR for circumferential AWE in unruptured IA was 1.86.

NLR has been evolving and used as an inexpensive peripheral blood marker for systemic inflammation in patients with acute ischemic and hemorrhagic stroke (21, 22, 27). Our study confirmed that NLR was significantly higher in patients with ruptured IAs than those with unruptured IAs, as shown in previous studies (28, 29). The elevated NLR at admission has been shown to predict the unfavorable functional outcome, delayed cerebral ischemia, in-hospital mortality, and rebleeding in patients with aneurysmal subarachnoid hemorrhage (23, 27, 29–31). An elevated NLR was also associated with aneurysm size in patients with unruptured IAs (29).

Notably, the relationship of NLR as a peripheral blood inflammatory marker with AWE was assessed in this study. We found that NLR at admission and aneurysm size was two predictors for circumferential AWE in patients with unruptured IA. Recent studies suggested wall enhancement as a sign of local inflammation within the aneurysm wall can be used as an imaging tool to assess the instability of unruptured IA (6, 9, 11, 12, 14, 15, 17, 25). The specificity of circumferential AWE to distinguish the instability of IA was higher than focal AWE (8, 25, 32). Moreover, circumferential AWE was correlated with the symptoms of sentinel headaches and third nerve palsy caused by unruptured IAs (7). The clinical value of NLR in unruptured IA, especially in aneurysms smaller than 7 mm, has been found in our study. The increased level of inflammatory cytokines in peripheral blood was associated with aneurysm size in both unruptured IA and thoracic aortic aneurysms (29, 33). Larger aneurysm size was more likely to rupture without preventive treatment. The elevated NLR was associated with circumferential AWE on HR-VWI, which might become a useful peripheral blood marker for assessing IA instability.

Our study provides more evidence for the association of blood biomarkers with AWE in unruptured IA. Recent studies have shown a strong correlation between blood inflammatory chemokines and gene expression in the aneurysm sac with AWE in unruptured IA (19, 34). Some gene expression in whole blood from the lumen of unruptured IA, such as CCDC85B, was associated with aneurysm size and AWE on HR-VWI, which may be a good candidate marker for identifying higher rupture risk IA. Our study revealed that the cutoff value of NLR for circumferential AWE was 1.86. In a study of 5,000 healthy Chinese adults, the mean value of NLR in healthy adult males and females was 1.59 and 1.62, respectively (35). In our study, the median NLR of patients with unruptured IA with circumferential AWE was 2.15, significantly higher than that in healthy adults.

Recently, quantitative measurement of AWE on HR-VWI has been used to avoid the subjectivity of the readers (19, 24, 26). CRstalk using maximal SI may be a new tool to predict the instability of unruptured IA. Swiatek et al. found that unruptured IA with CRstalk ≥0.5 was considered “enhancing” for AWE. In a study of 19 patients harboring 22 unruptured IAs, there was a significant association of CRstalk ≥0.5 with decreased plasma concentration of the anti-inflammatory cytokine IL-10 in the aneurysm sac (19). In our previous study, the optimal cutoff value of CRstalk for circumferential AWE was 0.5 (24). We found that the level of NLR at admission and aneurysm size was associated with CRstalk ≥0.5 in 57 patients with 60 unruptured IAs in the current study. An elevated NLR was associated with quantitative measurement of circumferential AWE using CRstalk ≥0.5 in unruptured IAs.

This study has some limitations. First, a retrospective study from a single center with a relatively small sample size might bias for patient selection and statistics analysis. Second, although AWE may be related to the inflammation mechanism within the aneurysm wall, the exact reason for associating higher NLR in the peripheral blood with circumferential AWE is not apparent; whether NLR may predict aneurysm instability requires a follow-up study. Finally, due to the retrospective design, our assessment of infection at admission may be somewhat inadequate despite our exclusion criteria, which may bias the result. However, this study included NLR in the multivariate logistic regression analysis and found that NLR was independently associated with circumferential AWE, so the bias may be limited.

## Conclusions

The NLR as a valuable peripheral blood inflammatory marker is more often in the rupture status of IA and was independently associated with circumferential AWE on HR-VWI in saccular unruptured IA. The clinical value of NLR combined with circumferential AWE for predicting the rupture risk of IA is needed to further investigation in a large sample size.

## Data Availability Statement

The original contributions presented in the study are included in the article/supplementary material, further inquiries can be directed to the corresponding author/s.

## Ethics Statement

The studies involving human participants were reviewed and approved by Sun Yat-sen Memorial Hospital. The patients/participants provided their written informed consent to participate in this study.

## Author Contributions

Z-SS participated in the design of the study and revised the manuscript. X-BW wrote the initial manuscript. All authors critically reviewed and edited the manuscript, approved the final version, participated in the interpretation and collection of the data.

## Funding

Z-SS is supported by the National Natural Science Foundation of China (Grant Numbers: 81873752 and 81720108014) and the Science and Technology Planning Project of Guangzhou City (Grant Number: 201704020166).

## Conflict of Interest

The authors declare that the research was conducted in the absence of any commercial or financial relationships that could be construed as a potential conflict of interest.

## Publisher's Note

All claims expressed in this article are solely those of the authors and do not necessarily represent those of their affiliated organizations, or those of the publisher, the editors and the reviewers. Any product that may be evaluated in this article, or claim that may be made by its manufacturer, is not guaranteed or endorsed by the publisher.
